# Spatially Multiplexed Speckle on 1D Sensors for High-Speed 2D Sensing Applications

**DOI:** 10.3390/s24113293

**Published:** 2024-05-22

**Authors:** Ricardo Rubio-Oliver, Martin Sanz, Michael Sigalov, Javier García, Yevgeny Beiderman

**Affiliations:** 1 Departamento de Óptica y de Optometría y Ciencias de la Visión, Facultad de Física, Universidad de Valencia, C/Doctor Moliner 50, 46100 Burjassot, Spain; ricardo.rubio-oliver@uv.es (R.R.-O.); martin.sanz@uv.es (M.S.); javier.garcia.monreal@uv.es (J.G.); 2 Faculty of Electrical and Electronics Engineering, Holon Institute of Technology, 52 Golomb Street, P.O. Box 305, Holon 5810201, Israel; michaelsi@hit.ac.il

**Keywords:** speckle pattern, 1D sensor, remote sensing, synthetic image

## Abstract

Speckle pattern-based remote vibration monitoring has recently become increasingly valuable in industrial, commercial, and medical applications. The dynamic and random nature of speckle patterns offers practical applications for imaging and measurement systems. The speckle pattern is an interference pattern generated by light scattered from a rough surface onto a remote plane. It is typically sensed using area scan cameras (2D), which are limited to framerates of 2–4 kHz and can only capture a small region of interest (ROI). In this work, we propose a technique that enables the capture of synthetic 2D speckle patterns using a 1D high-acquisition-rate sensor and a diffractive optical element (DOE) to produce image replicas. The multiple replicas are scanned by the 1D sensor simultaneously at different spatial positions. This method provides an ability to sense remote vibrations in all directions, contrary to the case with a simple 1D sensing system.

## 1. Introduction

Speckle pattern-based remote vibration monitoring has recently become very useful in industrial, commercial, and medical applications. Speckle pattern-based sensors are a type of optical measurement technique that utilizes the phenomenon of speckle patterns to gather information from surfaces or objects. Speckle patterns are random patterns that result from the interference of coherent light waves scattered from a rough surface or through a complex medium [1,2]. These patterns can be used to extract various types of information, such as displacement, deformation, and vibration. 

The basic principle behind speckle pattern-based sensors is that when a coherent light source, such as a laser, illuminates a surface, the scattered light creates a speckle pattern. Changes in the surface or object under investigation, such as vibrations, will cause the speckle pattern to change as well. 

One of the common techniques for speckle usage is the method of LASCA (laser speckle contrast analysis) [3]. In a broader sense, a dynamic speckle analysis is frequently used, based on the statistical properties of speckle patterns. Statistical analysis of speckle patterns has the potential to identify spatial areas where changes or activity occur at a slower or faster rate within the object. Various applications of this approach have been documented across fields such as medicine, biology, industry, and food quality assessment [4,5,6,7,8,9]. Sometimes, speckle imaging is also associated with ghost imaging [10].

Recently, a novel approach to the analysis of the speckle pattern was introduced. Considering that the speckle pattern can be captured by using a defocused imaging technique, it is possible to extract the desired information that is connected to the vibration phenomena, while later it is possible to relate it with a specific industrial or medical parameter referred to the measured phenomena [11,12,13,14,15].

The dynamic and random nature of speckle patterns has practical applications in imaging and measurement systems. The speckle pattern is an interfered pattern on a remote plane; therefore, the speckle pattern can be sensed with 2D cameras. Modern cameras work with a limited framerate, consequently constraining the possibility of sensing fast time-varying vibrational phenomena. Usually, those cameras come with framerates that are in the range of 2–4 kHz over a small ROI (region of interest). The use of this small ROI has been proposed to record audio signals in real time [16], although, given the aforementioned speed limitations of 2D sensors, they were only able to record signals up to 1 kHz, which is insufficient for key applications such as human voice recording. Higher framerate cameras exist, although they come with a much higher cost and limited internal memory storage that makes it difficult to capture long recordings and obtain real-time processing. 

The usage of 1D sensors is widely implemented in scientific and industrial applications. 1D sensors have recently been much improved in their capability to produce decent signals beyond the visual wavelength range. The 1D sensors are used for wafer integration [17], nanoparticles monitoring [18], and breast imaging [19]. 

The substitution of 2D detectors with 1D detectors holds significance beyond just the visible frequency range, where the need for fast recording arises. This issue also arises in the THz frequency range, where 2D detectors remain prohibitively expensive. To address this challenge, researchers are exploring diverse solutions, with the utilization of 1D line scanning as a promising compromise between single-pixel line scanning and expensive 2D detectors [20,21].

Recently, some 1D sensor recording techniques were proposed that provide much higher acquisition rates [22]. However, this technique is very sensitive to the directionality of the movement of a remote object (and as a result, the movement of speckle pattern over the sensor). If it does not coincide with the orientation of the 1D sensor, it does not produce any good reconstruction of the signal.

In this work, we propose a technique that makes it possible to capture 2D speckle patterns using a 1D sensor with a high acquisition rate. We introduce a diffractive optical element (DOE) just before the sensor plane in the visible range. DOE is an important element of the proposed system. 

The basic principal operation of the DOE is to passively manipulate light by redirecting and focusing it through the division and mutual interference of a light wave, contrary to refractive elements that achieve the same through changes in refractive indices [23]. 

The basic idea of our work is to make spatial multiplexing of speckle pattern over the 1D sensor with the DOE, while each replica is being captured at a different relative spatial location, therefore providing a possibility for synthetic reconstruction of the 2D pattern. The synthetic 2D pattern can be reconstructed from 1D sensor capture and processed as it would be originally produced by the 2D sensor, thus overcoming the directionality problem imposed by a 1D sensor (without the proposed multiplexing).

In this paper, we present a theoretical background, the method description, and experimental results. We also discuss some practical implementation issues, while comparative analysis versus the optimal recordings is analyzed. 

## 2. Theoretical Explanation

The speckle pattern is a physical phenomenon that relates to the self-interference patterns formed by the interaction of coherent light waves coming from a rough object. Coherent light is characterized by its consistent frequency and phase relationship between different points in space and time. When coherent light encounters a rough or diffusing surface, such as a rough paper, a textured wall, or a biological tissue, the light waves undergo random phase shifts and interference, resulting in a speckle pattern. This effect can be also referred to as self-interference [1,2,3].

Speckle patterns appear as a random distribution of bright and dark spots. The size and contrast of the speckles depend on factors such as the wavelength of light, the distance from the surface, and the spot size on the remote object Equation (1).

**Figure 1:**
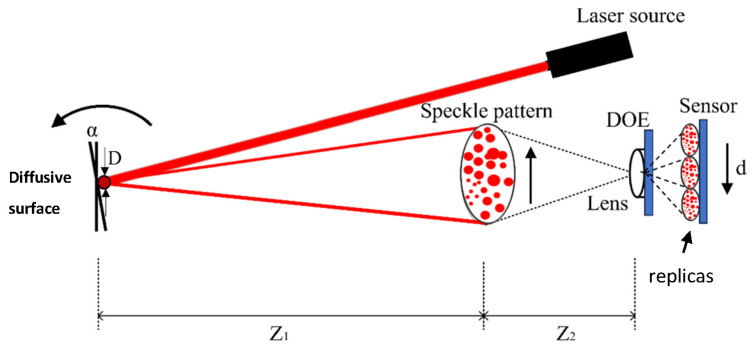
A schematic drawing of the experimental setup. The red color refers to the laser illumination.

Our configuration includes a projection of a laser beam and observation of the movement of the back-reflected pattern (the secondary speckle pattern) that is created on a sensor plane. In our configuration, the detection is obtained via a fast-imaging camera that observes the temporal intensity fluctuations of the defocused imaged speckle pattern and their trajectory Figure 1. To allow correlating the trajectory with the movement of the speckle patterns, we had to properly defocus our imaging lens [11].

Practically, defocusing switches the working point of our system to the far-field regime. There are three types of movements of the remote object: lateral, axial, and rotational. The lateral movement of the object will cause a proportional lateral movement of the speckle pattern over the sensor plane. However, the impact of this movement in the far field will be negligible. The axial movement will not impact the speckle pattern much either, as the object vibration amplitude along the axis is negligible compared to the object–camera distance. However, rotational movement of the surface plane on the object will cause a linear phase shift of the wavefront, while the speckle pattern will move laterally on the sensor plane (note: far-field regime). The lateral shift of the speckle pattern can be calculated by various techniques [24,25,26,27,28], and remote vibration movement can be reconstructed. 

The speckle size on the sensor is given by Equation (1):

(1) $$\delta x=\frac{\lambda Z_{1}}{D}\;\frac{1}{M}$$

where λ refers to the wavelength of the illumination, $Z_{1}$ is the object to focus plane distance, *D* is the diameter of the spot on the object plane, and *M* is the magnification of the optical system. 

The conversion of the angle of the rotation of the remote surface to the displacement of the speckle pattern on the camera sensor, *d*, is given by Equation (2):

(2) $$d=\frac{\alpha Z_{1}}{M}\;$$

where α is the angle of rotation at the object’s surface. The calculation for the focal length of the optical system required for obtaining a size of *K* pixels per speckle spot at the sensor plane is given as follows:

(3) $$F=\frac{K\Delta xZ_{2}D}{Z_{1}\lambda }$$

where Δ*x* is the pixel size and $Z_{2}$ is the imaging plane to lens distance. 

As we mentioned before, the speckle pattern moves laterally over the sensor plane when the remote object is vibrating. A way to reconstruct the remote vibration profile is to find the speckle pattern shift between the images subsequently captured by the sensor. We chose to perform a spatial cross-correlation over the two subsequent frames and find this shift by tracking the location of the max amplitude on the resulting correlation image. Therefore, the relative shift (*px*) between the two adjacent frames $I_{1}$ and $I_{2}$ will be:

(4) $$px=Loc[argmax\left\{Corr\left(I_{1,\;}I_{2}\right)\right\}]$$

where *Corr*() is the cross-correlation operator and *Loc*[] is the location extraction operator in the x-y plane of the resulting image. 

The *Corr*() is a cross-correlation operator that can be mathematically described as:

(5) $$Corr\left(I_{1,\;}I_{2}\right)=C\left(r,c\right)=\sum_{u=-h}^{h}\sum_{v=-h}^{h}I_{1}(r+u,c+v)I_{2}(u,v)$$

where $I_{1,}{\;I}_{2}$ are the input images of a size (2*h* + 1) × (2*h* + 1), and (*r*, *c*) are the spatial coordinates of the resulting image *C*, while (*u*, *v*) are the spatial indexes under the sum operator. 

An additional key element of the proposed system is a diffractive optical element (DOE). In our work, we use a linear multiplexer with five replicas, which are replicated onto the sensor plane Figure 1. The key reason for using the DOE is to multiply the spatial patterns and scan them with the 1D sensor in different spatial row positions all in one moment (by a single capture). This will provide us with the capability to synthetically reconstruct the 2D image with a faster framerate (as 1D sensors are much faster than the 2D), without losing the capability of two-directional sensing.

## 3. Description of the Method 

This work aims to replace a 2D sensor with the 1D sensor without losing the capability of two-directional sensing, while dramatically increasing the resulting acquisition framerate. To perform the task, we propose to add a diffractive optic element (DOE) at the position near the lens sensor plane Figure 1. The operation of the DOE is to multiplicate the pattern spot image over the sensor plane. 

**Figure 2:**
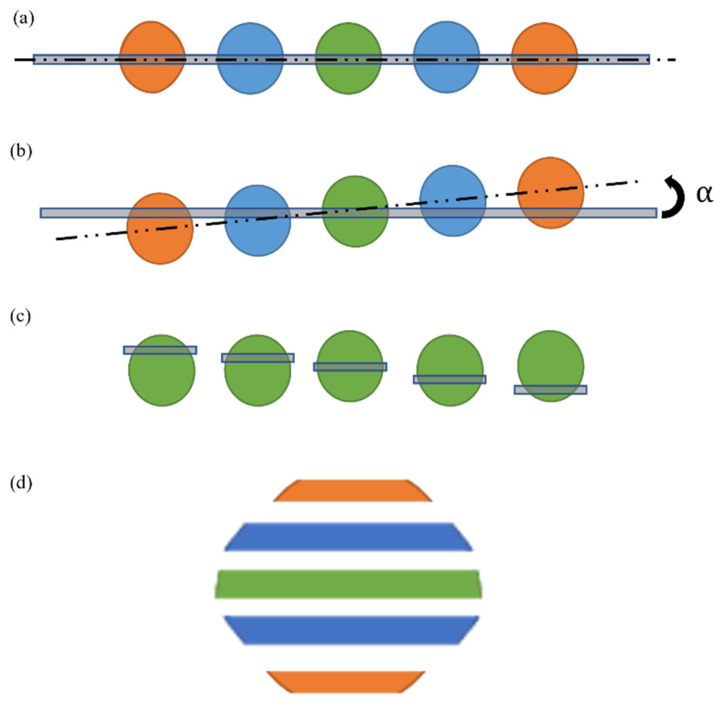
A multiplication principle of DOE in the system and the scanning scheme. (**a**) Sensor and DOE aligned: the five replicas and the 1D sensor line. (**b**) DOE is rotated by angle α: the sensor intersects each replica at a different height location. (**c**) The synthetic 2D scenario that is analogous to scanning the spot at five different height locations. (**d**) Scheme of how a synthetic spot is reconstructed from the combination of the information obtained at each replica from (**b**). Please note that each of the replicas is encoded by a different color (for the sake of clarity).

For such a purpose, we introduced a DOE that provides 5 replicas of the original pattern spot (which is sufficient for the proof of concept). When those 5 replicas are projected onto a linear sensor properly, we can reconstruct a single 2D spot of 5 lines of height. The condition to achieve this is to rotate the projection in a way that each replica of the spot will appear vertically displaced by one single pixel Figure 2. 

If the sensor is aligned with the direction of the DOE replicas, all the information captured by the sensor in each of the replicas remains the same Figure 2a. However, if the DOE is rotated by an angle α, the projection onto the sensor of each of the replicas will be different; in particular, it provides information from different locations of each spot Figure 2b. If the rotation is properly calibrated, one-pixel shift displacement is achieved between the replicas, allowing scanning of 5 consecutive areas of the spot along the direction perpendicular to the sensor Figure 2c. Of course, the one-pixel shift is not perfectly aligned as it is diagonal to the spot orientation; however, it will provide a slanted version of the 2D pattern. Later, we synthetically reconstructed the 2D image spot by adding the acquired lines from all the spots in a vertical direction Figure 2d. Please note that in the picture, each of the replicas is encoded by a different color (for the sake of clarity). However, in the real case, all the replicas maintain the same monochrome color (owing to laser illumination). 

To gain an advantage concerning 2D framerate, the 1D sensor framerate should be greater than the 2D. In practice, it is feasible to obtain a 20 kHz framerate from 1D sensors (which are widely available on the market) vs. 2 kHz for 2D sensors with a limited ROI. Therefore, a practical gain in the framerate can easily reach a factor of 10. 

### 3.1. Experimental Setup

**Figure 3:**
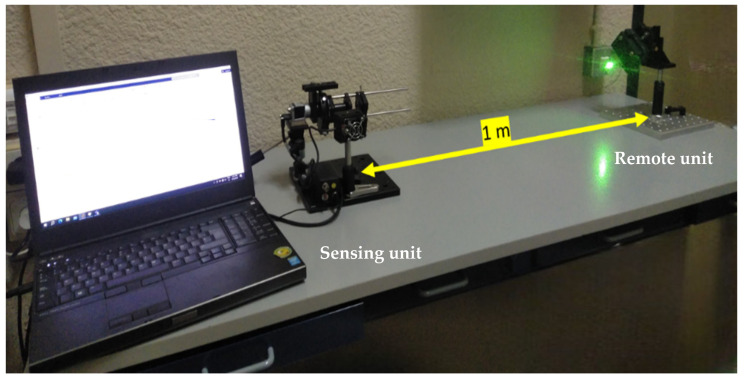
The experimental setup.

**Figure 4:**
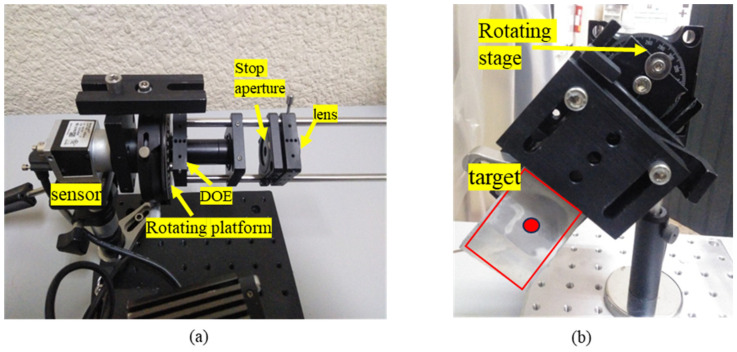
(**a**) The sensing unit of the system. (**b**) The remote vibrating unit. The vibration surface (target) is marked by a red rectangle. The piezoelectric actuator presses on the back side of the target in the location of the red spot marked to produce the tilting of the surface.

The setup consists of the laser and the optical system comprising a 2D camera, lens, DOE, and a rotation stage Figure 3. The camera was connected to a laptop computer used for storage of the captured frames and processing Figure 4a. On the other side of the system (remote unit), we prepared a controlled movement stage to produce different types of remote object vibrations Figure 4b.

The sensing unit of the system comprises:

1. 100 mm focal lens to project the speckles spot onto the sensor—(Newport, 25 mm Dia. × 100 mm FL, MgF2 Coated, Achromatic Doublet Lens)
2. Stop (variable diaphragm) to allow us to tune the diameter of the spot projected on the sensor. (Thorlabs variable stops SM1D12C).
3. DOE provides 5 replicas of the spot. (Holoeye DE-R 263)
4. Rotating platform. (PRM1, Thorlabs Inc., Lafayette, CO, USA)
5. Camera Sensor—(ACA 1300–200 um, Basler AG, Ahrensburg, Germany)
6. Laser—CNI laser, Power 40 mW, 532 nm, collimated. Class IIIb laser.

**Figure 5:**
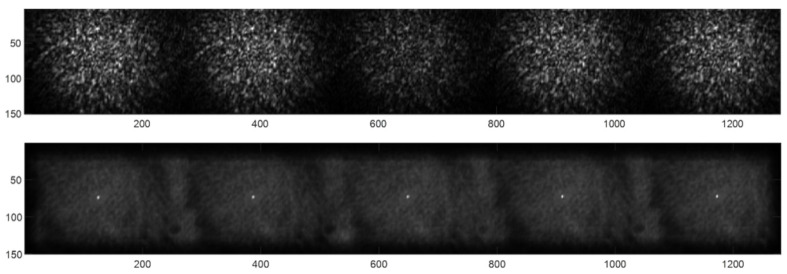
(**Upper image**): the image shows a captured frame with 5 replicas (with the lower half of the spot). (**Lower image**): the cross-correlated image showing the registration of the spot ROIs’ centers at the location of the correlation peaks.

All parts, as a block, are mounted on a high-precision rotation mount. One can accurately rotate the image on the sensor to achieve a proper rotation of the spot replicas on the sensor. The optimal angle of the rotation is achieved once one pixel in the vertical shift between the replicas is obtained Figure 5.

The remote vibration test unit was constructed with a controlled vibrating surface, which provides accurate displacement by a piezoelectric actuator. The entire unit was designed to provide almost pure 1D tilting. It is attached to a rotation stage to select the orientation of the tilting to perform tests at different orientations of the tilt. The specifications of this unit are:

1. Piezoelectric actuator—(45-1090, APC International Inc., Mackeyville, PA, USA).
2. Rotating stage—(RP01, Thorlabs Inc., USA)

The proof-of-concept setup of our method uses a 2D sensor. The main rationale for this choice, instead of the proposed 1D sensor, is to have the possibility of comparing the results with a conventional 2D sensor system. The 2D sensor can provide, by selecting a single row in the detector, a perfect analogue of the 1D sensor. 

To establish the correct alignment, we rotated the imaging system until we observed that the consecutive spots were displaced by only one pixel. This was easily achievable because the imaging system was mounted on a micrometric rotating mount. The captures were then processed, and the region of interest (ROI) of the first spot was selected. Subsequently, the following spots were detected by cross-correlation between them. This process enabled us to determine the relative shift between the ROIs due to the rotation of the imaging system Figure 5. 

In the upper image of Figure 5, the captured frame with 5 replicas (with half of the spot) is shown. The lower image of Figure 5 shows the result of cross-correlating the ROI of one of the spots with a zero-padded version of the whole image. The correlation peaks in the lower image indicate the ROI center per each spot. For this example, the centers’ locations in the *y*-axis are 69, 70, 71, 72, and 73, respectively. Therefore, a proper alignment was found, as a relative displacement between subsequent spots is of one single pixel.

### 3.2. Software Implementation and Performance Measurement

Using a 1D sensor camera provides an acquisition rate that can reach 20 kHz. This is the acquisition hardware limitation of the experiment. If we want to measure the movement of a surface during the grabbing in real-time, we should make sure to have software that processes the images and produces the result in real time (software and processing hardware limitation), meaning that every single image should be processed at most by 1/fps time. 

**Figure 6:**
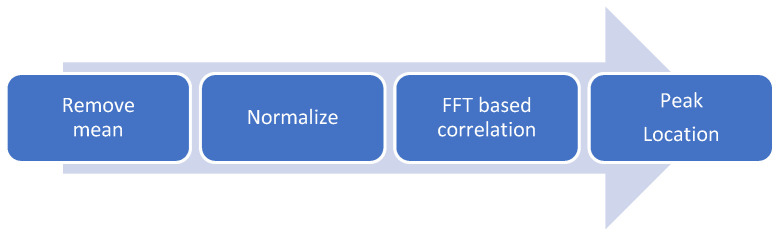
The flowchart of the software implementation.

For this experiment, a C++ [29] program was developed, calculating the shift of the speckle pattern using the correlation method as mentioned above (5). However, it was implemented by using the properties of correlation in the Fast Fourier Transform (FFT) to improve the time performance. The flowchart of the software code is presented in Figure 6. Please note that, for better processing, we first removed the mean and normalized the image; then we used FFT-based correlation, and finally located the correlation peak to extract the pattern shift.

The time performance was measured using EasyProfiler Software (version 2.1). Measurement times are shown in Table 1. The calculations were performed on a laptop with an AMD Ryzen 7 4800 H processor and 16 GB of RAM. The time needed for the whole process is 5 μs (microseconds), which enabled us to reach theoretical computational capability up to 200 kHz. Thus, we ensured that this processing is not the system bottleneck.

## 4. Results and Discussion 

Once the setup was constructed and tested, we captured a sequence of frames with the spot replicas. Later, we synthetically constructed a 2D spot by applying the technique described previously in Figure 2. 

**Figure 7:**
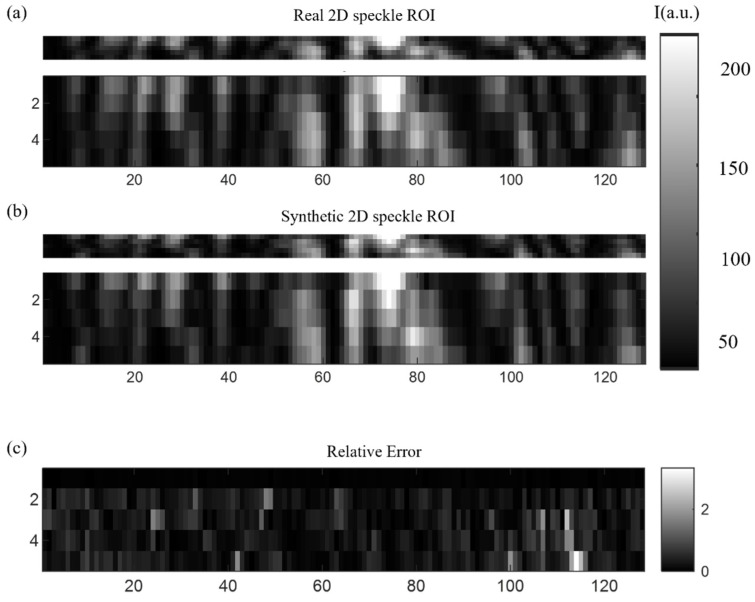
Comparison between the real 2D ROI and synthetically constructed one from a 1D combination of the multiplexed spots. The upper thin images in (**a**,**b**) are the images with the 1:1 axis ratio, while the lower one has been augmented in the vertical direction to facilitate the visualization. (**a**) Real 2D spot. (**b**) Synthetic 2D spot. (**c**) A relative error between (**a**) and (**b**) (dimensionless).

In Figure 7, we show a comparison between the real 2D ROI and the synthetically constructed one from the 1D combination of the multiplexed spots. The upper thin images in Figure 7a,b are the images with the 1:1 axis ratio, while the lower one has been augmented in the vertical direction to facilitate the visualization. Figure 7a is the real 2D spot captured by the sensor, while Figure 7b is a synthetic 2D spot. Figure 7c shows a relative error between the Figure 7a and Figure 7b. The relative error between the real and reconstructed images is low for most of the pixels, although some isolated pixels show a big discrepancy, even though the average relative error is about 0.2 (out of 256, owing to the 8-bit pixel depth).

**Figure 8:**
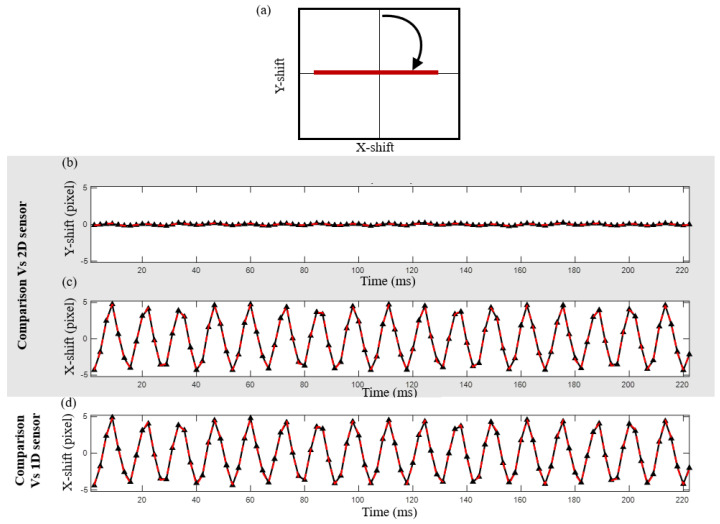
Experimental results for a tilt at 90 degrees from the Y−direction (mechanical tilt axis along the *X*-axis). (**a**) Parametric scheme of the movement (Y vs. X). (**b**) Measured vertical movement (*Y*−axis) from the 2D sensor (black solid line) vs. our method (the dashed red line). (**c**) Measured horizontal movement (*X*−axis) from the 2D sensor (black solid line) vs. our method (the dashed red line). (**d**) Measured horizontal movement (*X*−axis) by 1D sensor reconstruction (black line) versus our method.

**Figure 9:**
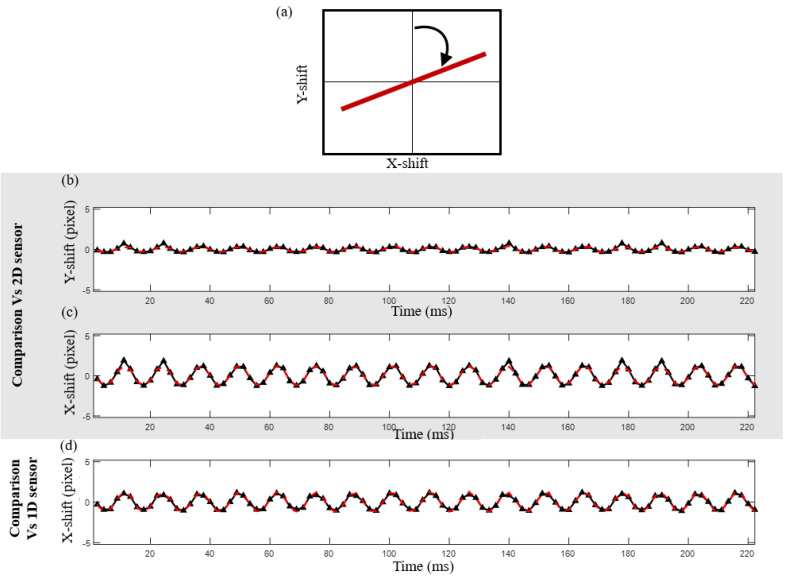
Experimental results for a tilt at 75 degrees from the Y−direction. (**a**) Parametric scheme of the movement (Y vs. X). (**b**) Measured vertical movement (*Y*−axis) from the 2D sensor (black solid line) vs. our method (the dashed red line). (**c**) Measured horizontal movement (*X*−axis) from the 2D sensor (black solid line) vs. our method (the dashed red line). (**d**) Measured horizontal movement (*X*−axis) by 1D sensor reconstruction (black line) versus our method.

**Figure 10:**
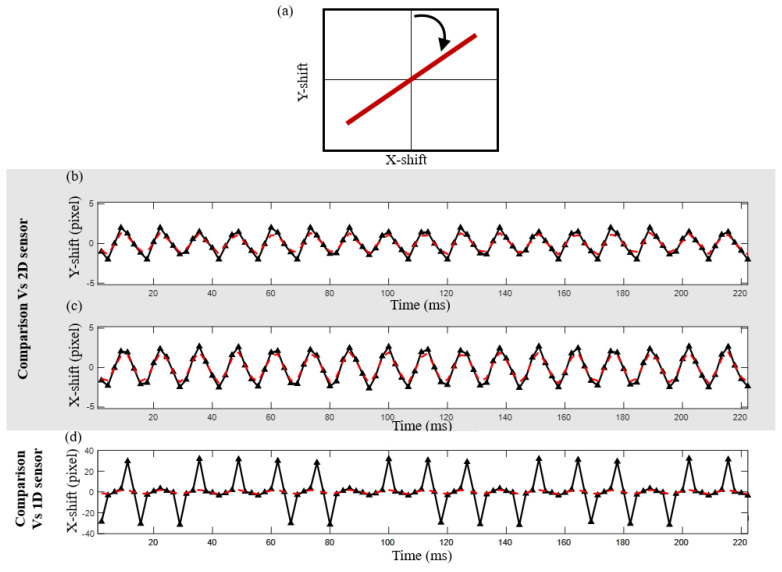
Experimental results for a tilt at 60 degrees from the Y−direction. (**a**) Parametric scheme of the movement (Y vs. X). (**b**) Measured vertical movement (*Y*−axis) from the 2D sensor (black solid line) vs. our method (the dashed red line). (**c**) Measured horizontal movement (*X*−axis) from the 2D sensor (black solid line) vs. our method (the dashed red line). (**d**) Measured horizontal movement (*X*−axis) by 1D sensor reconstruction (black line) versus our method. Note that the 1D direct reconstruction in X fails for many displacements.

A vibrating remote object was put to the test Figure 4b. Various scenarios were introduced and tested: a scan was performed between 0- and 90-degree angles with respect to the *Y*-axis. Angle selection was motivated to find a point when the signal would be dramatically distorted for the case of pure 1D sensing. In our case, we worked on rotations of 10, 20, 40, 60, 75, and 90 degrees to span all the possible positions with a reasonable step between the iterations. In Figure 8, Figure 9 and Figure 10, one can see a series of tests that were conducted to check the different states of directionality of object vibration. The tilt direction was selected at different angles, ranging from horizontal (tilt perpendicular to the *Y*-axis) to vertical (tilt parallel to the *Y*-axis). The upper window (Figure 8a) shows a reconstructed proportional displacement of the remote object in the X-Y plane. In this case, the surface was vibrating mostly in the horizontal direction (*X*-axis). As for the graph in Figure 8b, one can see a comparative graph of the relative movement of the remote object obtained from the 2D sensor (the black solid line) and the synthetic 2D spot, our method (the dashed red line), reconstructed in the *Y*-axis direction of the speckle pattern at the sensor plane. Figure 8c is the same as 8b but was obtained in the *X*-axis direction. Figure 8d represents the case for a 1D sensor (without applying our method for obtaining the 2D speckle) single-spot reconstruction of movement in the sensor direction (*X*-axis)—the black line—versus our synthetic 2D reconstruction from the 1D sensor, the dashed red line. In this case, as the movement was mostly in the *X* direction, it could be correctly recovered by employing a 1D sensor without the need to use our proposed method to generate 2D speckles.

Figure 9 and Figure 10 show the same results as in Figure 8, except for the case when the target surface vibrates at 60 and 75 degrees from the Y direction, respectively. In the last case, the purely 1D approach suffers from correlation loss, making it impossible to obtain a proper signal from a 1D sensor without adding our method to generate synthetic 2D speckles (Figure 10d). However, the results obtained from the proposed method show good agreement with the ones obtained using a 2D sensor (Figure 10b,c). 

In Table 2, one can see the resulting average error between the obtained reconstructed displacement profiles (Figure 8, Figure 9 and Figure 10) versus the real 2D sensor (for a variety of angles of vibration with respect to the Y direction). Obtained errors were generally below 10%, being in most cases about 5% for the Y direction (which would be perpendicular to the 1D sensor, thus being impossible to extract by just the 1D sensor). Similar results were obtained for the X direction, although generally with lower values, as expected. Additionally, the last column shows the results without using the proposed method, i.e., just working in pure 1D sensor mode (results are only available for the X direction as it is 1D). At the 60-degree and lower angles, we obtained huge errors for this purely 1D case, showing the effect of the correlation losses produced as the speckle pattern moved out of the sensing area. Note that the 1D direct reconstruction in X failed for many displacements, despite the sensed line being horizontal. On the contrary, by employing our proposed method, not only did we obtain information about the movement along the Y direction, but we also preserved the signal for the X direction while avoiding the correlation loss (loss of information).

The amplitude of the speckle moment movement (between the two adjacent frames) is limited by the number of pixels in each direction. The most limiting factor is the Y-direction (the reconstructed axis from the replicas). In our case it is only five replicas, meaning that the max amplitude should be less than half of this in order to not lose the correlation between the frames. The other direction (*X*-axis) moment movement is much less constrained as it is limited by two factors: the pixel count in the 1D sensor divided by the number of replicas. In our case, it is 1280/5 = 256 pixels. This is more than enough for any practical use if the sampling rate is properly adjusted for a given application. 

To improve the *Y*-axis movement reconstruction ability, one needs to introduce more replicas; however, it will limit the *X*-axis image size accordingly (to prevent replicas from overlapping after the DOE). The energetic efficiency of the sensor should be considered as well. As more replicas are produced, less light energy is left per spot (which can be critical in fast-rate applications). Therefore, a proper assumption of the possible movement of a remote surface should be considered per application to design the right image size vs. the number of replicas and the sensor efficiency.

## 5. Conclusions

In this work, we implemented a novel approach toward fast rate sensing of 2D speckle pattern movement utilizing 1D sensors. This was achieved by spatially multiplexing the 2D spot over the 1D sensor, allowing it to scan the spot for different heights in a single shot. This approach enables the use of faster framerate sensing without the problem of the directionality loss inherent to 1D sensing. This allows the recovery of 2D information on the speckle movement, as well as preventing the corruption of the signal due to correlation losses (produced when speckles move out of the sensing area). Both the theoretical explanation and experimental results are discussed. Additionally, a comparative analysis versus optimal recordings is presented. 

In future work, we would like to consider adding wavelength multiplexing techniques along with the proposed one. 

## Figures and Tables

**Table 1:** Performance measurement of the C++ program.

Process	Elapsed Time (Average)
Frame process (whole process)	5 μs
Remove Mean and Normalize	1 µs
Correlation	2 µs
Shift calculation	0.5 µs

**Table 2:** Resulting average error for different angles of vibration of the target with respect to the vertical direction.

Rotation Angle (°)	2D Synthetic Error *Y*-Axis (%)	2D Synthetic Error *X*-Axis (%)	1D Error *X*-Axis (%)
90	4.90	0.75	0.80
75	4.70	2.41	4.72
60	7.14	6.10	131.90
40	12.21	5.71	100.01
20	2.71	5.91	106.95
10	5.51	8.64	209.30

## Data Availability

The original contributions presented in the study are included in the article, further inquiries can be directed to the corresponding author.
